# H3K27me3 immunostaining is diagnostic and prognostic in diffuse gliomas with oligodendroglial or mixed oligoastrocytic morphology

**DOI:** 10.1007/s00428-021-03134-1

**Published:** 2021-06-24

**Authors:** Serena Ammendola, Nicolò Caldonazzi, Michele Simbolo, Maria Liliana Piredda, Matteo Brunelli, Pietro Luigi Poliani, Giampietro Pinna, Francesco Sala, Claudio Ghimenton, Aldo Scarpa, Valeria Barresi

**Affiliations:** 1 Department of Diagnostics and Public Health, Section of Anatomic Pathology, University of Verona, Policlinico G.B. Rossi. P.le L.A. Scuro 10, 37134 Verona, Italy; 2 Department of Translational and Molecular Medicine, Pathology Unit, University of Brescia, Brescia, Italy; 3 Department of Neurosciences, Unit of Neurosurgery, Hospital Trust of Verona, Verona, Italy; 4 Department of Neurosciences, Biomedicines and Movement Sciences, Institute of Neurosurgery, University of Verona, Verona, Italy; 5 Department of Pathology and Diagnostics, University and Hospital Trust of Verona, Verona, Italy; 6 ARC-NET Research Centre, University and Hospital Trust of Verona, Verona, Italy

**Keywords:** Astrocytoma, Oligodendroglioma, H3K27me3, 1p/19q codeletion, Recurrence

## Abstract

Oligodendroglioma is defined by *IDH* mutation and 1p/19q codeletion. The latter is mutually exclusive to ATRX immunohistochemical loss and has been recently associated with the loss of H3K27me3 immunostaining. We aimed to assess the diagnostic and prognostic value of H3K27me3 immuno-expression in diffuse gliomas with oligodendroglial or mixed oligoastrocytic morphology. H3K27me3 immunostaining was performed in 69 diffuse gliomas with oligodendroglial (n = 62) or oligoastrocytic (n = 7) morphology. The integration with routinely assessed *IDH* mutations, ATRX immunostaining, and 1p/19q codeletion classified these cases as 60 oligodendroglial and 9 astrocytic. H3K27me3 was lost in 58/60 oligodendrogliomas with retained (n = 47) or non-conclusive (n = 11) ATRX staining, 3/6 *IDH*-mutant astrocytomas with ATRX loss, and 3/3 *IDH*-wt astrocytomas. H3K27me3 was retained in 2/60 oligodendrogliomas with retained ATRX, and in 3/6 *IDH*-mutant astrocytomas, two of which had lost and one retained ATRX. The combination of H3K27me3 and ATRX immunostainings with *IDH* mutational status correctly classified 55/69 (80%) cases. In *IDH*-mutant gliomas, ATRX loss indicates astrocytic phenotype, while ATRX retention and H3K27me3 loss identify oligodendroglial phenotype. Only 14 (20%) *IDH*-mutant cases with retained ATRX and H3K27me3 or inconclusive ATRX immunostaining would have requested 1p/19q codeletion testing to be classified. Furthermore, H3K27me3 retention was associated with significantly shorter relapse-free survival (*P* < 0.0001), independently from *IDH* mutation or 1p/19q codeletion (*P* < 0.005). Our data suggest that adding H3K27me3 immunostaining to the diagnostic workflow of diffuse gliomas with oligodendroglial or mixed morphology is useful for drastically reducing the number of cases requiring 1p/19q codeletion testing and providing relevant prognostic information.

## Introduction

The 2016 World Health Organization (WHO) classification of gliomas, by integrating histopathological features with molecular alterations, classifies astrocytic tumours based on the mutational status of *IDH1/2* and *H3K27M genes*, while oligodendrogliomas are defined by the co-occurrence of *IDH1/IDH2* mutation and codeletion of whole chromosomal arms 1p and 19q [1]. This classification is prognostically informative, as oligodendrogliomas *IDH*-mutant and 1p/19q codeleted have the best prognosis among diffuse gliomas, while *IDH-*wt astrocytic tumours the worst [2]. In addition, diffuse gliomas with 1p/19q codeletion are more likely to respond to chemotherapy [3, 4].

*IDH* mutations in gliomas occur more frequently at residue p.R132 of *IDH1* and residue p.R172 of *IDH2* [5]. About 90% are *IDH1* R132H mutations that can be detected using a commercially available antibody against the mutant epitope [6], while 10% occur in other sites of *IDH1* or *IDH2* genes and can be currently identified only by DNA mutational analysis [2].

The WHO classification does not indicate a specific method to test 1p/19q codeletion but recommends that the assay should be able to detect whole-arm chromosomal losses [1], as the codeletion is mediated by a balanced whole-arm translocation of chromosomes 1 and 19 followed by the loss of one of the two derivative chromosomes composed of 1p and 19q [7]. Fluorescent in situ hybridization (FISH) is the most common method to assess 1p/19q codeletion [7]. However, this technique has shortcomings. First, it is unable to discriminate between complete and partial deletions of 1p and 19q because commercial probes hybridize to a minimal part of the chromosome arms, at 1p36 and 19q13 loci [8, 9]. Second, it cannot establish with certainty 1p/19q codeletion in the context of imbalanced aneuploidy, polyploidy, or polysomy [7, 9]. Other molecular methods, such as PCR-based LOH analysis, have higher specificity, but are more labour-intense and require a non-neoplastic control [7]. Therefore, the identification of surrogate markers of 1p/19q codeletion based on immunostaining may facilitate the differential diagnosis between astrocytoma and oligodendroglioma in routine practice.

In this context, it has been reported that the majority of *IDH*-mutant astrocytomas of all grades harbour truncating mutations in *ATRX*, which are mutually exclusive to 1p/19q codeletion and can be detected by the immunohistochemical loss of ATRX protein [2, 10, 11]. Thus, ATRX immunostaining has been suggested as an alternative low-cost marker to differentiate *IDH*-mutant astrocytomas from oligodendrogliomas [2, 10, 11]. Nonetheless, a proportion of *IDH*-mutant astrocytic tumours retain ATRX immunostaining [2, 12–14], and ATRX immunostaining has also been reported as non-conclusive in 11.5% of cases [14].

The immunohistochemical loss of histone 3 trimethylated in lysine 27 (H3K27me3) has been proposed to differentiate astrocytic from oligodendroglial neoplasms: diffuse gliomas can be classified astrocytic if retaining H3K27me3 expression, and oligodendroglial when showing H3K27me3 loss in association with mutated *IDH* and retained or non-conclusive ATRX staining [14]. This proposal stemmed from the finding of the immunohistochemical loss of H3K27me3 in 25/26 *IDH*-mutant and 1p/19q codeleted oligodendrogliomas and its retention in 120/135 astrocytomas [14]. A following study on 101 gliomas confirmed the significant association between H3K27me3 loss and 1p/19q codeletion but questioned its sensitivity and specificity [15].

We analysed H3K27me3 immunohistochemical expression in 69 diffuse gliomas with oligodendroglial or mixed oligoastrocytic morphology with the aim to clarify its diagnostic and prognostic potential, by assessing its correlation with 1p/19q codeletion and patients’ recurrence-free survival (RFS).

## Materials and methods

### Cases

We reviewed all cases operated between January 2014 and March 2020 at the University Hospital of Verona, Italy, and morphologically classified oligodendroglioma (OD), anaplastic oligodendroglioma (AOD), oligoastrocytoma (OAS), anaplastic oligoastrocytoma (AOAS), or glioblastoma with oligodendroglial component (GBM-O). A total of 69 cases, 62 with oligodendroglial (33 ODs and 29 AOs) and 7 with mixed morphology (4 OAS, 3 AOAS), were included in this study. All cases were classified according to WHO 2016 criteria [1]. None of the tumours was *H3.3* K27M mutant.

### Ethics

The study was approved by the local Ethics Committee (Comitato Etico per la Sperimentazione Clinica delle province di Verona e Rovigo; Prot. n. 11335, 02/26/2019) and performed in accordance with the ethical standards laid down in the 1964 Declaration of Helsinki and its later amendments.

### Clinical data

Information on tumour localization, extent of surgical resection, and development of recurrence was retrieved using clinical records. Recurrence was defined by means of computerized tomography or magnetic resonance imaging as identification of tumour growth at the site of previous surgery, or as a volumetric increase of tumour residue in case of subtotal or partial surgery. RFS was defined as the length of survival to the detection of a recurrent tumour.

### Immunohistochemistry

All cases were immunostained using antibodies against IDH1-R132H (clone H09, Dianova, GmbH, Germany; dilution 1:200), P53 (clone DO-7, Leica Biosystems, Newcastle, UK; prediluted), ATRX (Polyclonal; Life Science Sigma, St Louis, MO, USA; dilution 1:750), and H3K27me3 (clone C36B11, Cell Signaling Technology, Danvers, MA, USA; dilution 1:200), by means of an automated immunostainer (Leica Biosystems, Newcastle, UK).

ATRX expression was considered (i) retained, when nuclear staining was observed in both normal (endothelium, neurons) and neoplastic cells; (ii) lost, when staining was absent in neoplastic cells and present in normal cells; and (iii) non-conclusive, when staining was absent in both normal and neoplastic cells.

P53 immunostaining was assessed and scored as previously reported, and only cases with strong staining in at least 10% of neoplastic cells were rated p53 positive [16].

H3K27me3 immunohistochemical expression was classified according to Peckmezci et al. [15]: (i) retained, when nuclear staining was seen in ≥ 5% neoplastic cells; (ii) lost, when staining was absent in > 95% neoplastic cells and present in internal positive controls (endothelium, neurons); and (iii) non-conclusive, when staining was absent in both normal and neoplastic cells.

### IDH1/2 mutational analysis

In cases with negative immunostaining for IDH1-R132H, we assessed *IDH1/2* mutational status. Briefly, neoplastic cellularity was enriched to at least 70% by manual microdissection of 10 consecutive 4 μm sections. DNA was purified using the QIAamp DNA FFPE Tissue Kit (Qiagen) and qualified as reported [17]. *IDH1* and *IDH2* were amplified by PCR, and both strands were sequenced using the ABI PRISM 3500 Genetic Analyzer (Applied Biosystems). PCR conditions were (1) denaturation at 95 °C for 5 min; (2) 40 cycles at 95 °C/30 s, 58 °C/30 s, and 72 °C/30 s; and (3) elongation step at 72 °C/5 min. Primers used were IDH1-F CCATCACTGCAGTTGTAGGTT, IDH1-R GCAAAATCACATTATTGCCAAC, IDH2-F TGCAGTGGGACCACTATTATC, and IDH2-R GTGCCCAGGTCAGTGGAT.

### Chromosome arms 1p/19q codeletion analysis

FISH was used to assess 1p/19q codeletion. For each case, two consecutive 3-μm sections were processed with LSI 1p36/19q13 Dual-Colour Probe Sets assay (Vysis/Abbott, Molecular Europe, Wiesbaden, Germany), following manufacturer’s protocol. Slides were examined by Olympus BX61 fluorescence microscope equipped with a ×100 oil immersion objective and a triple band pass filter for simultaneous detection of Spectrum Orange, Spectrum Green, and DAPI signals. Two hundred non-overlapping nuclei containing a minimum of 2 reference probe signals were counted.

Cases were classified as (i) 1p/19q codeleted, when showing two reference probe signals (1q and 19p) and one target probe signal (1p and 19q) in at least 50% cells; (ii) non-codeleted, when having two reference probe signals (1q and 19p) and two target probe signals in > 50% cells; and (iii) imbalanced, when > 50% cells had a ratio of reference to probe signal different from 2/2 or 2/1 [18].

Cases with imbalanced 1p/19q status or *IDH*-wt and1p/19q codeleted at FISH analysis were further analysed using loss of heterozygosity (LOH) analysis. In detail, LOH was performed using tumour and normal DNA pairs extracted from paraffin-embedded tissue sections and evaluated by PCR-based LOH assays. Allelic loss analysis was performed using the microsatellite markers D1S508, D1S199, and D1S2734 on chromosome 1p, and D19S412, D19S112, and D19S219 on chromosome 19q. Forward primers were synthesized either with fluorescent tag FAM or HEX. PCR products were subjected to electrophoresis on an Applied Biosystems 310 automated DNA sequencer (Applied Biosystems, Italy) and fluorescent signals analysed using GeneScan software (Applied Biosystems). Allelic imbalance was evaluated by comparing PCR products from tumour and normal DNA. Peak height ratio was calculated, and allelic imbalance resulted from the ratio of normal to tumour signal (N1/N2 over T1/T2). LOH was assigned for values less than or equal to 0.5 and 1.5. Procedures were repeated three times and allelic losses assigned only upon consistency of the findings.

### MGMT promoter methylation analysis

The methylation status of O-6-methylguanine-DNA methyltransferase (MGMT) promoter was assessed in all cases. Briefly, 200 ng of DNA was incubated using sodium bisulphate included in EpiTect Plus FFPE Bisulfite Kit (Qiagen) and analysed using pyrosequencing through MGMT Plus kit (Diatech). The assay performs a quantitative analysis of the percentage of methylation of each of the 10 CpG islands located on chr10. Samples were stratified in four groups according to the extension of methylation status that has been related to clinical outcome: unmethylated (< 9%), low (range 9–20%), medium (21–35%), and high level (over 35%) [19].

### Statistical analysis

Considering only 66 *IDH*-mutant tumours, we calculated the sensitivity [true positive/(true positive + false negative)] and specificity [true negative/(true negative + false positive)] of ATRX+ vs ATRX+/H3K27me3−, for the identification of oligodendrogliomas.

Considering all the 69 tumours, we also calculated the sensitivity and specificity of ATRX− vs H3K27me3+ for the identification of astrocytomas.

RFS was assessed by the Kaplan-Meier method, with the date of primary surgery as the entry data and length of survival to the detection of a recurrent tumour as the end point. The Mantel-Cox log-rank test was applied to assess the strength of association between RFS and each of the parameters as a single variable. A probability (P) value less than 0.05 was considered significant. Statistical analyses were performed using MedCalc 12.1.4.0 statistical software (MedCalc Software, Mariakerke, Belgium).

## Results

**Fig. 1:**
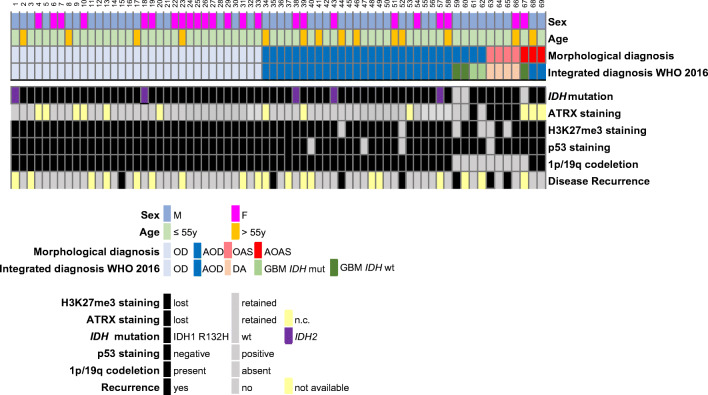
Clinical-pathological, immunohistochemical, and molecular features of 69 diffuse gliomas with oligodendroglial or mixed oligoastrocytic morphology. M, male; F female; OD, oligodendroglioma; AOD, anaplastic oligodendroglioma; OAS, oligoastrocytoma; AOAS, anaplastic oligoastrocytoma; DA, diffuse astrocytoma; GBM, glioblastoma; Wt, wild type; N.c., non-conclusive

The results are detailed in Fig. 1 and summarized in Supplementary Figure 1.

### Cases

All tumours were newly diagnosed gliomas with cerebral lobe localization, except for case 56, which was localized in the cerebellum. All neoplasms had gross total resection with the exception of case 15 that had subtotal resection. No patient had been treated with neo-adjuvant therapies. Forty-five patients were males and 24 females with mean age of 45 ± 11.6 years.

### *IDH* mutational status

Sixty-six of the 69 gliomas were *IDH*-mutant: 61 had *IDH1* R132H mutations detected by immunohistochemistry (IHC); 5 had *IDH2* mutations detected at sequencing analysis, including 2 ODs and 1 AOD with a R172K mutation and 2 AODs with a R172M mutation.

### 1p/19q codeletion by FISH and LOH analysis

Sixty of the 66 *IDH-*mutant gliomas were classified 1p/19q codeleted based on FISH and/or LOH analysis.

At FISH analysis of the 66 *IDH-*mutant cases, 57 had 1p/19q codeletion in > 50% cells and were classified 1p/19q codeleted; 5 were classified non-codeleted, and 4 cases had imbalanced 1p/19q. Of the 3 *IDH-*wt gliomas, 1 had imbalanced 1p/19q and 2 had 1p/19q codeletion.

The 7 cases with questionable 1p/19q status at FISH analysis, including 4 *IDH-*mutant and 3 *IDH-*wt, were further assessed by molecular LOH analysis (Table 1). Of the 4 *IDH-*mutant gliomas with imbalanced 1p/19q, 3 were codeleted and 1 non-codeleted. All 3 *IDH*-wt gliomas resulted non-codeleted.

**Table 1:** Immunohistochemical and molecular features of 7 diffuse gliomas with 1p/19q status assessed with LOH after FISH analysis

Case	*IDH* status	ATRX staining	H3K27me3 staining	1p/19 codeletion		Morphological diagnosis	Integrated diagnosis (WHO 2016)
				FISH	LOH		
7	Mutant	Retained	Lost	Imbalanced	Present	OD	OD
26	Mutant	Retained	Lost	Imbalanced	Present	OD	OD
69	Mutant	n.c.	Lost	Imbalanced	Present	AOAS	AOD
61	Mutant	Lost	Lost	Imbalanced	Absent	AOD	GBM *IDH* mut
60	wt	Retained	Lost	Imbalanced	Absent	AOD	GBM *IDH*-wt
59	wt	Retained	Retained	Present	Absent	AOD	GBM *IDH*-wt
67	wt	n.c.	Lost	Present	Absent	OAS	GBM *IDH*-wt

*FISH* fluorescent in situ hybridization, *LOH* loss of heterozygosity, *OD* oligodendroglioma, *AOD* anaplastic oligodendroglioma, *OAS* oligoastrocytoma, *AOAS* anaplastic oligoastrocytoma, *GBM* glioblastoma, *wt* wild type, *n.c.* non-conclusive

### Integrated diagnosis

Based on the assessment of *IDH* mutational status and 1p/19q codeletion, the cases were classified as follows: 33 ODs and 27 AODs *IDH-*mutant and 1p/19q codeleted; 4 diffuse astrocytomas (DAs) *IDH-*mutant; 2 GBMs *IDH-*mutant; and 3 GBMs *IDH*-wt (Fig. 1 and Supplementary Figure 1).

### ATRX and p53 immunohistochemistry

**Fig. 2:**
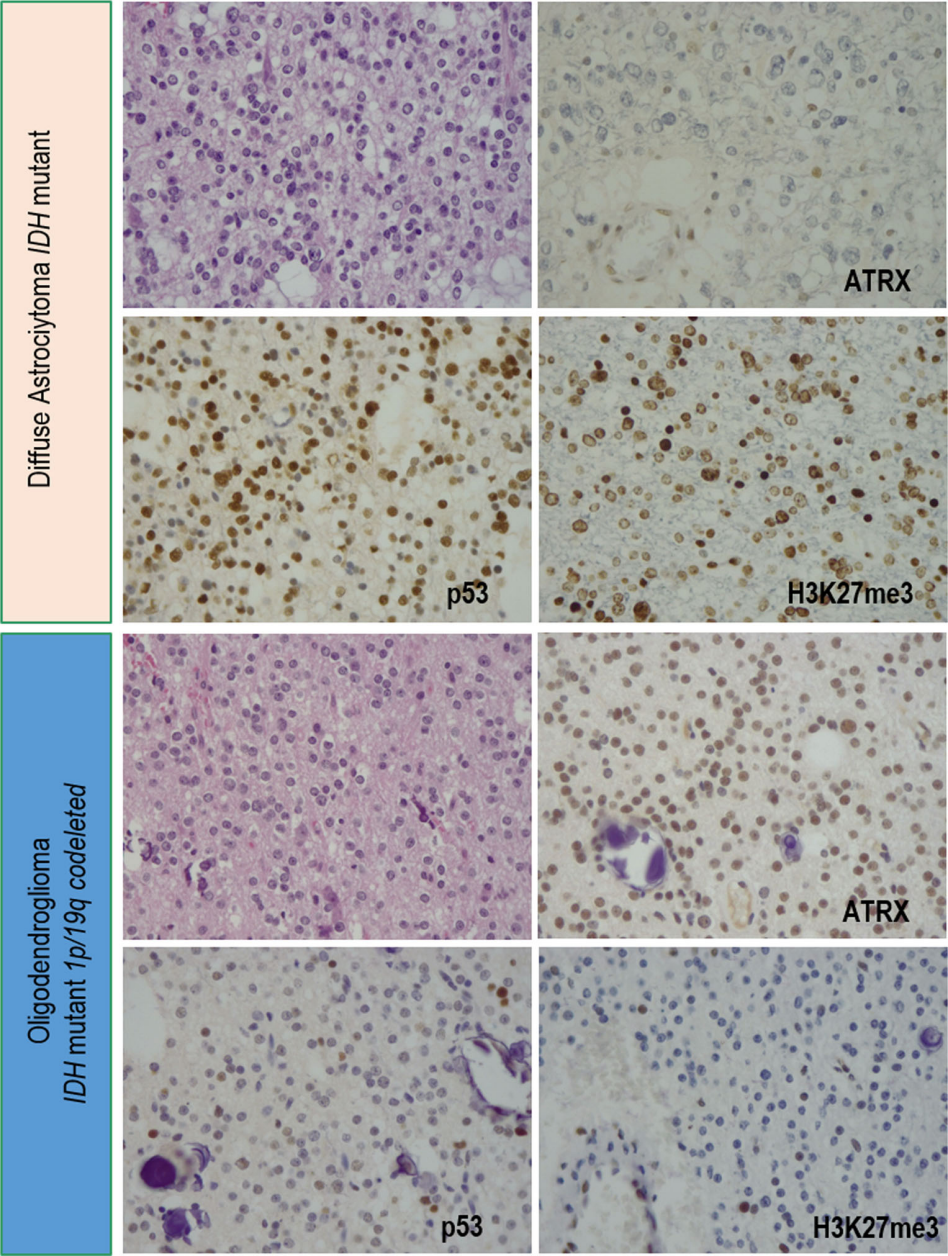
ATRX, p53, and H3K27me3 immunohistochemical expression in one astrocytoma and one oligodendroglioma. The *IDH*-mutant astrocytoma shows neoplastic nuclei with loss of ATRX, positivity for p53, and retained H3K27me3 immunostainings. The oligodendroglioma *IDH*-mutant and 1p/19q codeleted shows neoplastic nuclei with retained ATRX, scattered p53-positivity, and loss of H3K27me3 immunostaining

Of the 60 oligodendroglial (1p/19q codeleted) tumours, 49 retained ATRX immunohistochemical expression and 11 had a non-conclusive staining; 57 had a negative and 3 had a positive P53 staining; 58 lost and 2 retained H3K27me3 expression (Figs. 1 and 2; Supplementary Figure 1).

Of the 9 astrocytic tumours, 5 lost ATRX, 3 retained its expression, and 1 had non-conclusive staining; 8 were P53 negative and 1 was positive; 4 lost and 5 retained H3K27me3 expression (Fig. 1 and Supplementary Figure 1).

### H3K27me3 immunohistochemistry

H3K27me3 loss was significantly associated with the retention of ATRX immuno-expression (*P* = 0.025), 1p/19q codeletion (*P* = 0.0001), and negative P53 (*P* = 0.0027) (Supplementary Table 1).

### *MGMT* promoter methylation analysis

*MGMT* promoter was methylated in 58/60 oligodendroglial tumours (5 low, 21 intermediate, and 32 high level), in 4 *IDH*-mutant astrocytic (2 intermediate and 2 high level), and in 2 *IDH*-wt (1 low and 1 intermediate level). *MGMT* promoter methylation was more frequent in gliomas with H3K27me3 loss, but statistical significance was not reached (Supplementary Table 1).

### Sensitivity and specificity of ATRX and H3K27me3 immunostaining

In *IDH-*mutant tumours, ATRX+ was 100% sensitive and 87% specific to oligodendrogliomas, while ATRX+/H3K27me3− was 96% sensitive and 100% specific (Supplementary Table 2).

ATRX immunohistochemical loss was 62% sensitive and 100% specific for astrocytic tumours, while H3K27me3+ was 44% sensitive and 97% specific (Supplementary Table 3).

*IDH*-mutant tumours with non-conclusive ATRX staining were excluded from the calculation.

### Proposed diagnostic algorithm

**Fig. 3:**
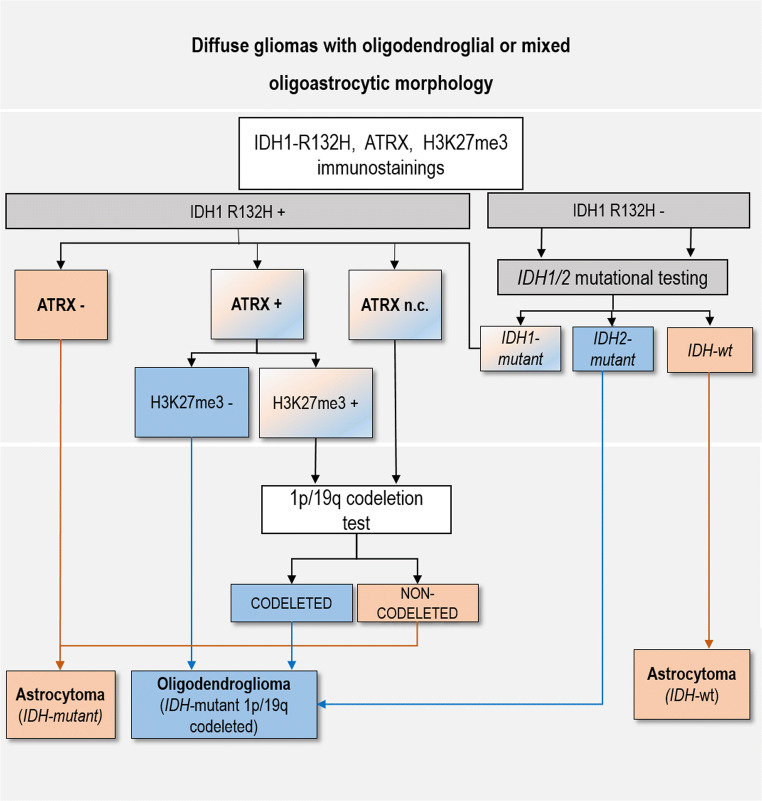
Proposed diagnostic algorithm for diffuse gliomas. Diffuse gliomas are classified starting with the assessment of IDH1 R132H, ATRX, and H3K27me3 immunostainings. Cases with ATRX loss are classified astrocytic, while those positive for IDH1 R132H and with retained ATRX and lost H3K27me3 stain are classified oligodendroglial. Cases with negative IDH1 R132H are tested for other *IDH1/2* mutations: the *IDH-*wt is classified astrocytic, while the *IDH*-mutant with retained ATRX and lost H3K27me3 is classified oligodendroglial. The assessment of 1p/19q codeletion is reserved to *IDH-*mutant tumours with retained ATRX and H3K27me3 immunostainings or non-conclusive ATRX. Using this approach in the present study, 55/69 cases would have been classified correctly without 1p/19q codeletion testing, which would have been needed in only 14 cases

Based on our results, we propose the diagnostic algorithm illustrated in Fig. 3. IDH1-R132H, ATRX, and H3K27me3 immunostainings should be assessed first. Then, based on the immunohistochemical results, (i) cases with ATRX loss are classified astrocytic; (ii) cases positive for IDH1-R132H with retained ATRX and lost H3K27me3 are classified oligodendroglial; and (iii) cases positive for IDH1-R132H with non-conclusive ATRX or retained ATRX and H3K27me3 should be tested for 1p/19q codeletion. Cases with negative IDH1-R132H staining should be analysed for *IDH1/2* mutations to complete the evaluation.

Applying this algorithm to our series, 47 (68%) cases would have been classified astrocytic or oligodendroglial using only immunohistochemistry for IDH1-R132H, ATRX, and H3K2727me3, 8 (12%) cases would have been resolved with *IDH1/2* molecular testing, while only 14 (20%) of the 69 cases would have requested the assessment of 1p/19q codeletion by FISH or PCR-microsatellite LOH analysis (Supplementary Figure 2).

### Recurrence-free survival analysis

Follow-up data were available for 50 patients. Seven patients had a recurrence, with a RFS ranging between 18 and 47 months (median: 30 months; inter-quartile range: 24–41 months). The remaining 43 patients were free of recurrence in a follow-up period ranging between 6 and 80 months (median: 33 months; inter-quartile range: 17.5–44 months) (Fig. 1).

Of the 7 gliomas that relapsed, 5 retained H3K27me3 immunohistochemical expression (*P* < 0.0001) (Supplementary Table 1).

**Fig. 4:**
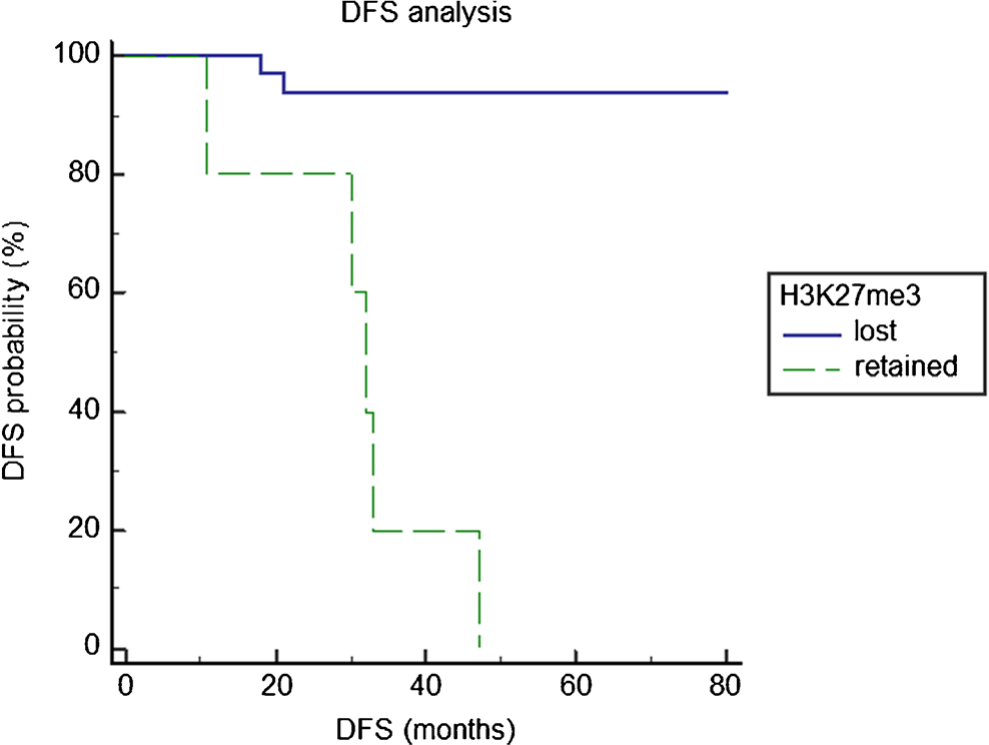
Recurrence-free survival (RFS) analysis of 55 patients with diffuse gliomas, according to H3K27me3 expression. The RFS of patients with a tumour showing H3K27me3 retention was significantly shorter than that of patients harbouring a tumour with H3K27me3 loss (*P* < 0.0001)

Patients harbouring a tumour with retained H3K27me3, *IDH-*wt, lack of 1p/19q codeletion, or grade IV had significantly shorter RFS (Fig. 4; Supplementary Table 4). Multivariate analysis, including *IDH* mutational status, 1p/19q codeletion, histological grade, and H3K27me3 staining as covariates, showed H3K27me3 immunohistochemical expression as the only significant and independent predictor of RFS (hazard ratio: 38; confidence interval: 2.9.1–513; *P* = 0.005) (Supplementary Table 4).

## Discussion

The diagnosis of oligodendroglioma requires the coexistence of *IDH1/2* mutation and 1p/19q codeletion [1]. This latter is assessed in most pathology laboratories by FISH, which is time and expertise demanding [7]. Thus, finding surrogate immunohistochemical markers of 1p/19q codeletion could avoid expensive and unnecessary tests [20].

The results of our study suggest that this can be achieved applying an algorithm that starts with the assessment of *IDH* mutations associated with ATRX and H3K27me3 immunostainings. This approach would reserve 1p/19q codeletion testing only to *IDH-*mutant gliomas with retention of both ATRX and H3K27me3 or non-conclusive ATRX immunostainings.

The proposed algorithm is based on our observation that ATRX loss was 100% specific to the astrocytic phenotype, which confirms the data repeatedly reported in the literature [2, 13, 21], and that the co-occurrence of retained ATRX and loss of H3K27me3 in *IDH-*mutant gliomas was 100% specific to oligodendroglial phenotype.

The utility of H3K27me3 immunostaining in the differential diagnosis between oligodendroglioma and astrocytoma was first reported by Filipski et al. [14], who suggested that H3K27me3 retention indicates astrocytic (non-codeleted) tumours with a predicted probability of 0.9995, while its loss in *IDH-*mutant gliomas with retained or non-conclusive ATRX stain is 100% specific for oligodendrogliomas [14].

Although the association between the loss of H3K27me3 immuno-expression and 1p/19q codeletion was confirmed in three independent studies [15, 22, 23], Peckmezci et al. found retained H3K27me3 in 7/28 (25%) oligodendrogliomas, and the co-occurrence of H3K27me3 loss and retained or non-conclusive ATRX staining in 7/74 (10%) *IDH-*mutant astrocytomas [15]. This latter study therefore questioned the assumptions of Filipski et al. that H3K27me3 retention can be used as the sole evidence against 1p/19q codeletion, and that the co-occurrence of H3K27me3 loss and retained or non-conclusive ATRX in *IDH-*mutant gliomas is specific to oligodendrogliomas [15]. The discrepancy between the results of Peckmezci et al. [15] and those of Filipski et al. [14] could be partially explained by the use of different working dilutions of the same anti-H3K27me3 antibody (clone C36 B11) they used (1:50 vs 1:200). Indeed, Kitahama et al. demonstrated that the higher the antibody dilution, the higher the specificity in recognizing oligodendrogliomas [23].

In the present study, we performed H3K27me3 immunostaining using the same antibody clone of Filipski et al. [14] and Peckmezci et al. [15] at the 1:200 working dilution used by Filipski et al. [14]. Our data confirm that H3K27me3 loss is significantly related to both 1p/19q codeletion and ATRX retention.

The protocol that we suggest differs from that of Filipski et al. in that the latter classifies all tumours with retained H3K27me3 as astrocytic, with no additional tests [14], while cases with H3K27me3 loss are tested for ATRX and *IDH* mutations, and those *IDH*-mutant with retained or non-conclusive ATRX are considered oligodendroglial [14]. Using the protocol of Filipski et al. on our series, 2 AODs would have been misclassified as astrocytomas as they retained H3K27me3, while using our proposed diagnostic flowchart, we would have correctly diagnosed as oligodendroglial or astrocytic all cases. In particular, 47 of 69 (68%) cases would have been solved with immunostaining for IDH1-R132H, ATRX, and H3K2727me3. Mutational analysis of IDH1-negative cases would have solved 8 (12%) additional cases; thus, 1p/19q codeletion analysis would have been limited to only 14 (20%) of the 69 diffuse gliomas with oligodendroglial or mixed morphology. Noteworthy, all our *IDH2*-mutant gliomas resulted oligodendroglial, reinforcing the reported association between *IDH2* mutations and 1p/19q codeletion in diffuse gliomas [24, 25].

In this respect, it is important to note that our study was limited to diffuse gliomas with oligodendroglial or mixed oligoastrocytic morphology, because *IDH-*mutant astrocytic-appearing tumours with ATRX loss can be confidently diagnosed as astrocytic without the need for 1p/19q codeletion testing [21]. Furthermore, the key role of morphology in guiding immunostaining and molecular tests is also highlighted by our study in which the morphological diagnosis was confirmed by the molecular data in 58/69 (84%) cases, while it was modified in 11 (16%) cases.

Reducing the need for 1p/19q testing reduces not only costs but also the pitfalls associated with it. The widely used FISH analysis has difficulties of interpretation in the case of imbalanced 1p/19q status and the impossibility to discriminate between complete and partial loss of chromosomal arms [7]. Despite high concordance with other molecular techniques, such as PCR-microsatellite LOH analysis, FISH has not only a lower sensitivity in detecting whole-arm 1p/19q losses [7] but also a lower specificity due to false positive astrocytomas with partial chromosomal losses [26, 27].

The limitations of FISH were also evident in our study, where its results were deceiving in 7/69 (10%) cases, including the interpretation of five cases with imbalanced 1p/19q and two *IDH*-wt false positive gliomas. The five gliomas with imbalanced 1p/19q status resulted codeleted (3 cases) or non-codeleted (2 cases) at PCR-microsatellite LOH analysis. Noteworthy, our protocol would have correctly identified 4 of these *5* cases with imbalanced 1p/19q using ATRX and H3K27me3 immunostainings, and only one case would have requested 1p/19q testing. The false positive results in the two *IDH*-wt gliomas are easily avoided as *IDH*-wt gliomas are diagnosed as astrocytic without further testing [1].

H3K27me3 stain also proved to be prognostically informative. Namely, retention of H3K27me3 was significantly associated with the development of recurrent disease and with a shorter RFS, independently from *IDH* mutational status and 1p/19q codeletion. Indeed, all 5 gliomas with retained H3K27me3 and only 2/45 with H3K27me3 loss recurred. This corroborates the previously reported association between H3K27me3 loss and a longer survival in 58 patients with diffuse gliomas [14], and demonstrates that in these tumours, H3K27me3 has a prognostic significance opposite to that found in meningiomas or ependymomas, where its retention associates with a better prognosis [28, 29].

In conclusion, the present study shows that the introduction of H3K27me3 immunohistochemistry to the diagnostic algorithm of diffuse gliomas with oligodendroglial morphology may be useful to predict 1p/19q codeletion status and provide relevant prognostic information. Our study strongly suggests that in *IDH*-mutant gliomas, ATRX loss indicates astrocytic phenotype, ATRX retention, and H3K27me3 loss indicate oligodendroglial phenotype, while retained or non-conclusive ATRX and retained H3K27me3 mandate for 1p/19q codeletion testing. Finally, the loss of H3K27me3 is associated with a significantly better prognosis, independently from 1p/19q codeletion and *IDH* mutational status.

## Data Availability

Data will be available upon request to the corresponding author.
